# Beware of the Zebra: Nine-year-old with Fever

**DOI:** 10.5811/cpcem.2019.5.42119

**Published:** 2019-07-01

**Authors:** Kathryn Lupez, Bryant Allen, Sean Fox, Margaret Lewis

**Affiliations:** Carolinas Medical Center, Department of Emergency Medicine, Charlotte, North Carolina

## Abstract

An otherwise healthy nine-year-old female who spoke only French presented with abdominal pain, vomiting, intermittent fevers, fatigue, and headache. She then quickly became febrile and altered requiring intubation. When treating a healthy child, the physician may initially develop a differential that includes common illnesses. Yet, as emergency medicine providers, we must be thinking about the “zebras” in order to not miss potentially deadly, curable diseases.

## CASE PRESENTATION

A nine-year-old female arrived to the emergency department (ED) with her parents who described a chief complaint for their daughter of “fever and vomiting.” They elaborated on the case and described that the patient had been experiencing diffuse abdominal pain, vomiting, intermittent subjective fevers, and decreased energy, with development of a mild headache earlier that day. She had normal bowel movements, no hematemesis, no bilious emesis, and no known sick contacts. Review of systems was negative for rash, ear pain, cough, chest pain, dysuria, and extremity pain. The patient had no past medical or surgical history, and she was current on her vaccinations. She was not on any daily medications and had no allergies to medications. Of note, the patient and her family members spoke only French and an interpreter was used throughout the examination.

Physical exam revealed an overall ill-appearing child. The vital signs were as follows: temperature of 98.5° Fahrenheit (F), heart rate of 140 beats per minute (bpm), blood pressure of 97/54 millimeters of mercury (mmHg), respiratory rate of 22 breaths per minute (BPM), and SPO_2_ of 100% on room air. In general, she was sleepy but arousable. She had dry mucous membranes. Her tympanic membranes were clear bilaterally. She had normal S1 and S2 heart sounds with no murmurs, rubs, or gallops. Her lungs were clear to auscultation bilaterally. Her abdomen was soft yet tender to palpation in all quadrants with noted splenomegaly. She was moving all four extremities equally. There was no rash noted on exam. Initial workup included a complete blood count, venous blood gas, complete metabolic panel, and urinalysis as documented in Table 1.

On re-examination, the patient had become completely unresponsive. Repeat vital signs revealed the following: a temperature of 102.5° F, heart rate of 160 bpm, blood pressure of 75/40 mm hg, respiratory rate of 30 BPM, and SPO_2_ of 92% on room air. She was subsequently resuscitated with an intravenous (IV) fluid bolus. We obtained central access and initiated vasopressors. The patient was then intubated for airway protection given her severely altered mental status.

A head computed tomography (CT) was obtained (Image). The radiologist read it as “No acute intracranial findings.” An abdominal and pelvic CT was obtained (Image), which the radiologist read as “Mild splenomegaly for age. Small volume ascites. No other acute intra-abdominal processes.” We performed a lumbar puncture and obtained a cerebral spinal fluid (CSF) sample as documented in Table 2. A confirmatory test was then sent from the ED and a diagnosis was made.

### Faculty Approach

This case describes an otherwise healthy nine-year-old female who presented to the pediatric ED with subjective fevers and abdominal pain for approximately one week. When treating a healthy child without significant medical history, one may initially apply the common adage “when hearing hoofbeats think horses” and develop a differential that includes common illnesses such as urinary tract infection or pyelonephritis, appendicitis or ruptured appendicitis, infectious gastroenteritis, flu-like illness or viral infection not otherwise specified, or even streptococcal pharyngitis. Fever and abdominal pain are extremely common complaints in the ED.

**CPC-EM Capsule**

What do we already know about this clinical entity?

*Malaria is known as one of the deadliest diseases in human history and still today, there are over 200 million cases and over 500,000 deaths annually, predominately in children.7*

What makes this presentation of disease reportable?

General illness and fever are common “hoof beats” heard in the pediatric emergency department yet as we become more interconnected than ever before, we need to “beware of the zebras”.

What is the major learning point?

There are three general phases of malaria including the incubation phase, “uncomplicated” malaria phase, and the “complicated” malaria phase.

How might this improve emergency medicine practice?

Review of the presentation of severe malaria is crucial in aiding emergency medicine providers in identifying an uncommon yet treatable deadly disease.

However, our patient had had no specific symptoms of dysuria or flank pain to suggest a urinary tract infection. Nor did she have sore throat or exudates to support strep pharyngitis. Also, the patient had normal bowel movements. Although an infectious gastroenteritis with dehydration was certainly possible, it was less likely given the presentation. Appendicitis was not supported by the timeline or physical exam. Ruptured appendicitis was considered given the duration of symptoms and diffuse abdominal pain; however, the CT abdomen and pelvis revealed splenomegaly and ascites, but no signs of ruptured appendicitis.

Further history and a thorough physical exam became critical. Further history indicated that in addition to a one-week time course the patient had begun to develop headaches. The physical exam was significant in that she was noted to be ill-appearing and sleepy, with an abdominal exam that was diffusely tender but not peritoneal and with splenomegaly present. Because fevers, headaches, and abdominal pain are all vague and common complaints, one must keep a broad differential and consider a rather expansive workup. In addition, the patient appeared to be falling off the proverbial cliff and becoming more critically ill in the ED.

Common causes of fever and abdominal pain need to be evaluated as well as fever and headache. Fever itself can lead to headaches, but fever and headache in an ill-appearing child should make one consider possible tick-borne illness depending on location and time of year, meningitis, encephalitis, and even possibly intracranial abscess. Despite the fully immunized status of the patient, this presentation would warrant neurologic imaging and a lumbar puncture.

A comprehensive metabolic panel and complete blood count along with urinalysis and venous blood gas were initially ordered given the initially stated complaint of abdominal pain, vomiting and fever. Normal urinalysis effectively ruled out urologic etiology of her symptoms. However, the remainder of the labs were remarkable for hyponatremia, increasing blood urine nitrogen and creatinine, pH of 7.1 with a remarkably elevated lactate, and pancytopenia with white blood count of 1.2, hemoglobin of 7.7, and platelets of 95.

As a patient rapidly declines in the ED, the astute physician will continue to listen to hoofbeats and think horses; at the same time, he or she must now pause and consider zebras, as well as prioritize resuscitative measures. Our patient was intubated, had central access established, and underwent head and abdominal CT imaging (Image). Abdominal CT imaging noted splenomegaly and small ascites, but no definitive causes of the patient’s symptoms. Normal head CT served to eliminate intracranial abscess, but meningitis and encephalitis were still potential causes and a lumbar puncture was performed. CSF was notable for clear fluid, slightly elevated opening pressure, high glucose, normal protein, and lymphocyte predominance. The white cells in the CSF were elevated at 17 per microliter but not reflective of a bacterial infection. The white cells, lymphocyte predominance, elevated glucose, and normal protein in the CSF analysis did not reflect a bacterial cause of the patient’s symptoms (Table 2).

Following appropriate resuscitation and attempted stabilization, it was critical to reflect back to the presenting symptom, physical exam findings, and known laboratory values. The patient presented with abdominal pain and splenomegaly, fevers and headaches, became acutely and severely ill, and had pancytopenia as well a profound elevation in lactate.

Sepsis, leukemia, and megaloblastic anemia all can cause a pancytopenia. Sepsis was certainly high on the differential given that the patient had fevers and became severely ill requiring the use of vasopressors. The patient herself never had any cough or shortness of breath to suggest pneumonia as cause of sepsis, nor any urologic complaints and had a normal pulmonary exam, no costovertebral angle tenderness, and a normal urinalysis. She also had a lumbar puncture performed with results that did not reflect bacterial meningitis. Other causes of sepsis, cholecystitis and bacteremia were less likely given that the patient was fully immunized and had no prior medical problems and no indwelling IV lines.

Acute cholecystitis in the pediatric population is rare. Risk factors for acute acalculous cholecystitis (more common in children) include trauma, burns, surgery, and severe systemic infection,^1^ of which this patient had none. Leukemia was also certainly on the differential as well in this patient with pancytopenia, unexplained fevers, and splenomegaly. She also had fatigue, which certainly fit the presentation of acute leukemia. Childhood leukemia is one of the most common childhood malignancies, and the patient had many signs and symptoms that supported this diagnosis – fevers, fatigue, splenomegaly, and pancytopenia. However, other symptoms that would have supported a diagnosis of acute leukemia – shoddy lymphadenopathy, easy bruising, recurrent infections, limb pain, and weight loss – were not present in this case.^2^ The patient’s mother gave no history of recurrent infections, bruising, anorexia, or any other vague symptoms prior to onset of illness; the patient was healthy and well. With megaloblastic anemia, one would expect a more chronic picture of fatigue, pallor, shortness of breath, and light-headedness, none of which correlated with the patient presentation.

Other causes of pancytopenia include immunosuppressive medications, meningitis, and familial causes. The patient had no history of receiving any immunosuppressive medications, no known family history, and the CSF results did not clearly support meningitis.

Pancytopenia can also be caused by a variety of other viral infections and even parasitic infections. A clear history of present illness and thorough physical exam should help include or exclude different infections. Viral causes may include parvovirus, human herpes virus 8, cytomegalovirus and Epstein-Barr virus. Common symptoms will include fevers, fatigue, headache, and throat pain. Although several of these symptoms corresponded to the patient’s symptoms, they did not offer a perfect fit. Finally, one must also consider systemic parasitic infections as the cause of the patient’s pancytopenia, and a clear history of potential exposures is critical.

As the patient and family spoke only French, all history was obtained through an interpreter. Although the French language is typically associated with France (and Bordeaux, brie, la Cote D’Azur, Paris, and Notre Dame), there are 29 countries in the world that identify as francophone, or French-speaking. France is the largest francophone country in terms of native speakers, followed by Canada. Interestingly, approximately half of the global French-speaking population lives in Africa, in countries including Congo, Cote d’Ivoire, Madagascar, Cameroon, Senegal, Burkina Faso, Mali, and Niger.^3^

Pancytopenia has a broad and potentially very serious differential including sepsis, acute leukemia, megaloblastic anemia, drug-induced, meningitis, viral, and parasitic causes. The patient herself was an otherwise healthy, French-speaking, young female with fever, abdominal pain and headaches now with an acute and severe deterioration in clinical condition with pancytopenia and a significant elevation in lactate.

Which diseases become “horses,” or common diseases, as opposed to “zebras,” or rare diseases, is relative to where the patient resides or travels. So, what if the “zebra” in this case were really a French-speaking horse from Africa? It would explain the patient’s vague symptoms and severe disease progression. We needed one final laboratory test to make this diagnosis.

## FINAL DIAGNOSIS

As the patient continued to decompensate in the ED, the providers knew further investigation was necessary to help save this child’s life given the lack of a definitive diagnosis. Due to concern for incomplete communication, despite use of a French interpreter, further history was gathered on re-examination. It was revealed that the patient and her family were from the Congo and had just arrived in the United States a few weeks earlier. The Centers for Disease Control and Prevention (CDC) malaria guidelines note that an estimated 216 million cases of malaria occurred in 2016 globally, with most cases in sub-Saharan Africa. Malaria itself can present with vague symptoms including fevers, abdominal pain, and fevers, and may progress to severe disease and even death if caused by *Plasmodium falciparum*.^4^

The conglomeration of symptoms including her fever, vomiting, altered mental status, splenomegaly, pancytopenia, multiple organ failure, and increased intracranial pressure led the providers to send one confirmatory test to make their diagnosis. This test was a peripheral blood smear, specifically a thick and thin smear, which revealed *P. falciparum* leading to a final diagnosis of cerebral malaria. From the ED, we contacted the 24-hour CDC hotline,^5^ which helped allocate the appropriate anti-malarial medication. The patient was started on a quinine drip and admitted to the pediatric intensive care unit. Remarkably, within four weeks she made a full recovery and returned home with her family.

### Beware of the Zebra

Many of us were taught the common aphorism in medical school: “when you hear hoofbeats, think horses not zebras.” When approaching a nine-year-old with fever, we hear these hoofbeat symptoms and tend to think of the typical diagnoses that are commonly seen in our pediatric population. Yet if we are not thinking about the zebras, we will miss this common presentation of a disease that is uncommon north of the equator, which could lead to high morbidity and possibly even mortality for patients

Malaria is known as one of the deadliest diseases in human history with speculations that it has contributed to the death of over half of all people who have ever lived.^6^ Still today, there are over 200 million cases and over 500,000 deaths annually, predominantly in children.^7^ Endemic regions of tropical and subtropical climates form a ring around our globe that is commonly referred to as the “malaria belt.”^8^ While most of our EDs are not located within this “malaria belt,” we have to be prepared. We are more interconnected then we have ever been with thousands of people traveling all over the world every day. Last year alone, our world tourism data was the highest yet with an annual traveler increase of 7%.^9^ Malaria, one of the deadliest diseases in human history, will inevitably be arriving soon to an ED near you.

Although there are many different *Plasmodium* species, each with its own nuances, there are three overarching phases that can help us understand the presentation of a patient with malaria.^10^ Phase 1 is the incubation phase. This is the time from disease exposure to symptom onset. This typically occurs when one is located in a geographical area at risk; surrounding female *Anopheles* mosquitos can flood malarial sporozoites throughout the blood stream with just one bite. From the time of the bite forward, one can be completely asymptomatic for any time between 7–30 days depending on the *Plasmodium* species.^10^ This long incubation period is dangerous given that our patients do not often think to tell us what they were doing a week ago, much less a month ago. We must beware of the zebra and collect a detailed travel history to make this “can’t miss” diagnosis in our patients.

Phase 2 is the “uncomplicated” malaria phase. The sporozoites mature in the liver to schizonts, which then mature to merozoites that readily infect red blood cells. Merozoites rapidly reproduce within the red blood cell until they rupture the red blood cell open, releasing even more merozoites to start the cycle all over again. Each time the merozoites rupture a red blood cell, an intense inflammatory reaction occurs including fever, chills, diffuse muscle aches, lethargy, gastrointestinal upset, and headaches.^10^ The reproduction and rupture of the red blood cell is typically cyclical in nature with the length between the reaction varying widely between each *Plasmodium* species. This may lead to a cyclical presentation of clinical symptoms. Yet because patients rarely follow textbook presentation, this can lead to a diagnostic dilemma, particularly in the months of December and January when these inflammatory symptoms can mimic common viral syndromes such as influenza. Yet we must remember to keep our differential broad and beware of the zebra.

Phase 3 is the “complicated” malaria phase. This occurs when the merozoites have replicated to the point of infiltrating just about every organ system. Merozoites can invade the central nervous system, leading to altered mental status, seizures, and extremely high intracranial pressures, also known as cerebral malaria. It can lead to liver failure and renal failure. Although rare, it can lead to a secondary hemophagocytic lymphohistiocytosis presenting as complete pancytopenia as seen in our patient.^11^ Systemically, it can present with profound hypotension, fever, and hypoglycemia. If you heard the hoofbeats of these systemic symptoms on any typical day in the ED, you would probably call this sepsis. The hoofbeats of fever with altered mental status may be mistaken as meningitis. The intra-abdominal organ failure might be pinned as some intra-abdominal catastrophe, or the pancytopenia as a new-onset malignancy, but beware of the zebra. If you weren’t thinking of this zebra, the complicated malaria phase has close to a 100% mortality without treatment.^12^

Thinking of zebras may afford clinicians the ability to pick up on some of the important clues and spur them to order that one crucial diagnostic test the peripheral blood smear. The thick peripheral blood smear will identify the parasitemia load and the thin smear will identify the *Plasmodium* species.^13^ Once a diagnosis is made, the 24-hour CDC malaria^5^ hotline should be contacted to ensure proper management as well as to expedite any appropriate antimalarials.

Remember that the world we live in is more interconnected than it has ever been. Malaria, one of the deadliest diseases in human history, is coming to your ED, perhaps as soon as your next shift. When you hear those hoofbeats, beware of the zebra.

## Figures and Tables

**Image:**
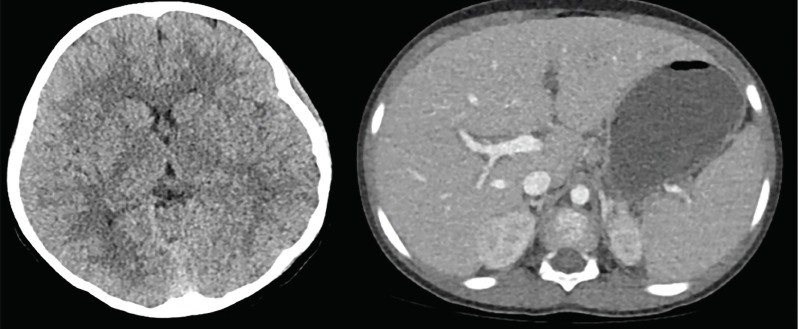
Computed tomography of the head (left) and abdomen and pelvis (right).

**Table 1:** Laboratory evaluation of child presenting with fever, abdominal pain, and vomiting.

Comprehensive metabolic panel	
Sodium	128 mmol/L
Potassium	3.7 mmol/L
Chloride	97 mmol/L
Bicarbonate	11 mmol/L
Glucose	75 mg/dL
Blood urine nitrogen	51 mg/dL
Creatine	1.4 mg/dL
Aspertate aminotransferase	34 IU/L
Alanine aminiotransferase	130 IU/L
Complete blood count	
White blood cells	1.2 Thousand/uL
Hemoglobin	3.7 g/dL
Platlets	95 Thousand/uL
Venous blood gas	
pH	7.1
Partial pressure carbon dioxide	29 mm Hg
Lactate	13.9 mmol/L
Urinalysis	
Leukocyte Esterase	Negative
Nitrates	Negative
White blood cells	0

*mmol,* millimole; *L,* liter;* mg,* milligram; *dL,* deciliter; *IU,* international unit;* uL,* microliter;* mmHg,* millimeters of mercury.

**Table 2:** Cerebral spinal fluid findings.

Cerebral spinal fluid	
Opening pressure	31cm H_2_O
Color	Clear
Protein	57 mg/dL
Red blood cells	1 count/mm^3^
White blood cells	15 count/mm^3^
Monocytes	25%
Lymphocytes	75%
Blasts	0%
No Xanthochromia	

*cm,* centimeter; *H_2_O,* water; mg, milligram; *dL,* deciliter;* mm,* millimeter.
